# A review on the oncogenesis of Merkel cell carcinoma: Several subsets arise from different stages of differentiation of stem cell

**DOI:** 10.1097/MD.0000000000033535

**Published:** 2023-04-14

**Authors:** Yueyang Zhu, Yuan Yin, Fuqiang Li, Zhiyuan Ren, Yaru Dong

**Affiliations:** a Department of Ophthalmology, The Second Hospital of Jilin University, Changchun, Jilin, China; b Department of Mechanical Engineering, University of Illinois Urbana Champaign, Champaign, IL.

**Keywords:** Merkel cell carcinoma, Merkel cell polyomavirus, oncogenesis, stem cell, ultraviolet

## Abstract

Merkel cell carcinoma (MCC), a rare primary cutaneous neuroendocrine neoplasm, is extremely aggressive and has a higher mortality rate than melanoma. Based on Merkel cell polyomavirus (MCPyV) status and morphology, MCCs are often divided into several distinct subsets: pure MCPyV-positive, pure MCPyV-negative, and combined MCC. MCPyV-positive MCC develops by the clonal integration of viral DNA, whereas MCPyV-negative MCC is induced by frequent ultraviolet (UV)-mediated mutations, that are characterized by a high mutational burden, UV signature mutations, and many mutations in *TP53* and retinoblastoma suppressor gene (*RB1*). Combined MCC consists of an intimate mix of MCC and other cutaneous tumor populations, and is usually MCPyV-negative, with rare exceptions. Based on the existing subsets of MCC, it is speculated that there are at least 4 stages in the natural history of stem cell differentiation: primitive pluripotent stem cells, divergent differentiated stem cells, unidirectional stem cells, and Merkel cells (or epidermal/adnexal cells). In the first stage, MCPyV may integrate into the genome of primitive pluripotent stem cells, driving oncogenesis in pure MCPyV-positive MCC. If MCPyV integration does not occur, the stem cells enter the second stage and acquire the ability to undergo multidirectional neuroendocrine and epidermal (or adnexal) differentiation. At this stage, accumulated UV-mediated mutations may drive the development of combined MCC. In the third stage, the stem cells differentiate into unidirectional neuroendocrine stem cells, UV-mediated mutations can induce carcinogenesis in pure MCPyV-negative MCC. Therefore, it has been speculated that several subsets of MCCs arise from different stages of differentiation of common stem cells.

## 1. Introduction

Merkel cell carcinoma (MCC), a rare primary cutaneous neuroendocrine neoplasm, is extremely aggressive and has a higher mortality rate than melanoma.^[1,2]^ In the United States, the annual incidence of MCC was 0.7/100,000 persons in 2013, and there has been an exponential global increase over the past 30 years.^[3,4]^ Risk factors for developing MCC include chronic ultraviolet (UV) exposure, immunosuppression, skin fires, and older age.^[5]^ MCC usually presents as an asymptomatic, rapidly growing, pink-red or violaceous, firm, solitary papule or nodule, typically on the head or neck region but also on the extremities or buttocks.

Histopathologically, 3 types of MCC have been described: trabecular, intermediate, and small cell types in the dermis. Generally, MCC tumors consist of a monotonous population of basophilic, small, round cells with hyperchromatic nuclei, scant cytoplasm, and indistinct nucleoli. Nuclear molding with finely dispersed chromatin, mitosis, and apoptotic bodies was frequently observed. These tumor cells tend to infiltrate deep tissues as well as lymphatic and vascular vessels. Early dissemination and metastasis often occur at the time of diagnosis.^[6]^ The dominant ultrastructural characteristics are round membrane-bound dense-core neurosecretory granules, approximately 100 nm in diameter, perinuclear whorls of intermediate filaments, and complex intercellular junctions.^[7]^

A classical immunohistochemical panel containing 5 markers, cytokeratin-20 (CK20), cytokeratin-7 (CK7), chromogranin A, neurofilament (NF), and thyroid transcription factor-1 (TTF-1), is commonly used for the diagnostic evaluation of MCCs.^[8]^ Most MCCs were CK20 positive, chromogranin A positive, NF-positive, TTF-1 negative, and CK7 negative. CK20 is a low-molecular-weight cytokeratin normally expressed in the gastrointestinal epithelium, urothelium, Merkel cells, and MCCs, the latter with a distinctive perinuclear dot-like pattern.^[9,10]^ CK7 and TTF-1 are commonly used to exclude metastasis from small cell lung carcinoma.

MCC is a unique cutaneous neuroendocrine tumor, and its histogenesis continues to be a topic of debate in the scientific literature. In this review, we summarize various existing opinions and speculate that several subsets of MCCs arise from different stages of differentiation of common stem cells.

## 2. Methods

A comprehensive search was performed using PubMed/Medline from inception to January 28, 2023. Our search queries were (“carcinoma, merkel cell” [MeSH Terms] AND “combined” [Text Word]) OR (“carcinoma, merkel cell” [MeSH Terms] AND “collision” [Text Word]) OR (“carcinoma, merkel cell” [MeSH Terms] AND “concurrent” [Text Word]). The inclusion criteria were that the articles should be in the English language and that the concurrent MCC tumor should be a primary cutaneous neoplasm. The reference lists of the articles were reviewed to identify additional citations.

## 3. Classification

Although MCC has historically been discussed as a singular entity, recent literature has often divided it into several distinct subsets based on the status of Merkel cell polyomavirus (MCPyV) and morphology, and the clinical importance of these subsets is under investigation.^[11]^

### 3.1. Stratified based on MCPyV status

In 2008, Feng et al reported a breakthrough in understanding MCC biology through their discovery of MCPyV, which was monoclonally integrated into the genome of tumor cells.^[12]^ MCPyV, a novel human polyomavirus, comprises a closed, circular, double-stranded DNA. Further studies confirmed that MCPyV is a ubiquitous virus that causes asymptomatic infections in the general population and is integrated into approximately 80% of MCCs.^[13]^ Two T antigens of MCPyV, small T and large T antigens, expressed in MCC, are involved in the transformation and proliferation of tumor cells.^[14]^ MCPyV is believed to be a carcinogenic agent, making MCC the only human cancer frequently caused by polyomaviruses.^[15]^

Based on MCPyV status, MCC can be divided into 2 broad categories: MCPyV-positive subsets, which represent the majority of cases, and MCPyV-negative subsets.^[16]^ Two subsets have different molecular characteristics; MCPyV-positive MCCs are associated with viral DNA integration and few mutations,^[17,18]^ whereas MCPyV-negative MCCs present a high mutational burden with UV signature mutations.^[19]^ Morphological differences also exist between the 2 subsets: MCPyV-positive MCCs are uniform tumor cells with round nuclei and less cytoplasm, whereas MCPyV-negative MCCs are more pleomorphic tumor cells with small and large irregular nuclei and more cytoplasm.^[20,21]^ Moreover, MCPyV-positive MCCs have a better survival prognosis than other subsets.^[22,23]^

### 3.2. Stratified based on morphology

Generally, MCCs are morphologically segregated into 2 groups: pure MCC, which refers to MCC occurring in isolation, and combined MCC, which consists of an intimate mix of MCC and other cutaneous tumor populations.^[24]^ MCC usually arises in association with other types of cutaneous neoplasms within the same lesion, including squamous cell carcinoma (SCC), Bowen disease, actinic keratosis, follicular cysts, trichoblastoma, basal cell carcinoma, and lentigo maligna, among others.^[21,25]^ MCC co-occurring with SCC (both in situ and invasive) is the most common, comprising 5% to 34% of all MCCs.^[9]^

Most pure MCCs are MCPyV-positive, and a small number are MCPyV-negative. Combined MCC is usually MCPyV-negative with rare exceptions. Combined MCC arises on chronically UV-exposed sites, often during immunosuppression.^[26]^ Pure MCCs are localized in the dermis with no epidermal involvement, whereas combined MCC often exhibit epithelial changes, such as ulceration and hyperkeratosis.^[27]^ Patients with combined MCC, comparing with those with pure MCC were older (median 76.5 vs 69 years) and had more nonmelanoma skin cancer (85% vs 25%), malignant extracutaneous tumors (25% vs 5%), and immunodeficient states (77% vs 35%). Patients with combined MCC had more metastases (77% vs 40%) and shorter survival (41 vs 54 months) than those with pure MCC.^[26]^ Combined MCC usually has a poor prognosis.^[28]^

## 4. Pathogenesis

The mechanism of tumorigenesis in MCCs differs between MCPyV-positive and -negative subsets. Immunohistochemical and molecular analyses suggested 2 modes of MCC pathogenesis: MCPyV-induced and UV-mediated pathways.^[29]^ MCPyV-positive MCC develops via the clonal integration of the virus, whereas MCPyV-negative MCC is induced by frequent UV-mediated mutations.^[23]^

### 4.1. MCPyV-induced oncogenesis

MCPyV DNA is clonally integrated into the host cell genome, leading to truncating mutations in the viral large T antigen gene before the helicase domain and the subsequent acquisition of an oncogenic program.^[16]^ The truncated large T antigen sequence prevents viral replication through the loss of helicase but allows for binding to the retinoblastoma suppressor gene (*RB1*) and inactivates it, which may play a role in initiating and/or maintaining transformation.^[21]^ Immunosuppression may facilitate the replication of MCPyV, which contributes to viral integration, mutagenesis, and carcinogenesis.^[30]^

### 4.2. UV-mediated oncogenesis

In MCPyV-negative MCCs, a higher mutational burden, UV signature mutations, and many mutations in *p53* and *RB1* have been well documented.^[31]^ The overall mutation burdens were 10.09 ± 2.32 and 0.40 ± 0.09 mutations per Mb in MCPyV-negative and -positive MCCs, respectively. A prominent UV signature pattern with C > T transitions, comprising 85% of the mutations, was observed in MCPyV-negative MCCs, whereas MCPyV-positive MCCs lacked a UV signature.^[18]^ *RB1* displayed the highest mutation rate, and *RB1* expression was absent in MCPyV-negative MCCs.^[32]^ Interestingly, *RB1* inactivation induces the expression of SOX2, a master transcription factor involved in Merkel cell development.^[33]^ In line with this, strong SOX2 positivity was observed in MCPyV-negative MCCs.^[32]^

## 5. Origin

The histogenesis of MCC continues to be a topic of debate in scientific literature.^[34]^ Several authors have suggested that MCC arises from Merkel cells.^[35]^ Other authors have suggested an origin from a primitive pluripotent stem cell that has the potential for divergent differentiation into keratinocytes or neuroendocrine cells.^[36]^

### 5.1. Hypothesis about MCC originated from Merkel cell

In 1972, Toker^[37]^ originally described and designated primary cutaneous neuroendocrine neoplasms as trabecular carcinomas, initially thought to be of sudoriferous or eccrine origin. Subsequently, ultrastructural identification of tumor neurosecretory granules similar to those of Merkel cells led to the eponym MCC.^[35]^

Merkel cells located within the basal cell layer of the epidermis are slow-acting mechanoreceptors because they are in contact with unmyelinated nerve fibers.^[2,38]^ Two theories have been proposed regarding the origin of Merkel cells. One possibility is that Merkel cells result from neural crest-derived cells of the amine precursor uptake and decarboxylation system,^[39]^ whereas others consider that it originated from embryonic epidermal stem cells.^[40]^ Merkel cells share the same immunohistochemical profile as MCC (positivity for cytokeratin and neuroendocrine markers) and have historically been regarded as the origin of MCC.^[36]^

However, arguments against the possibility of MCC arising from Merkel cells also exist. This is because the differentiated Merkel cells lack mitotic activity,^[41]^ are insensitive to oncogenic stimuli in mice,^[42]^ and have poor MCPyV infectibility.^[43]^

### 5.2. Hypothesis about MCC originated from epidermal or adnexal stem cell

Over the past 30 years, combined MCC, a distinct subset of MCC that co-occurs with other skin tumors in the same lesion, has been treated as a window into the histogenesis of all MCCs. Possible causes of combined MCC include the development of both components from common precursor cells or simultaneously from 2 separate precursor cells under a common oncogenic influence, such as chronic UV exposure.^[6,44]^

#### 5.2.1. Combined MCC derived from common precursor cells.

Combined MCC, most commonly MCC associated with SCC (both in situ and invasive), comprises 5% to 34% of all MCCs.^[9,26]^ The frequency of coexistence is higher than that attributed to chance associations. Many authors have regarded cases of combined MCC as evidence that the origin of all MCCs is a primitive totipotent or multipotent stem cell capable of neuroendocrine, glandular, and/or squamous differentiation, whether localized to the epidermis, dermis, or follicular epithelium.^[25,32,45,46]^

Combined MCC shares the same immunohistochemical staining with follicular pluripotent stem cells; therefore, follicular stem cells may be the possible origin of combined MCC and may undergo multidirectional differentiation into squamous, glandular, and neuroendocrine components.^[45]^ Squamous and glandular differentiation is often present in MCC, which prompted authors to suggest an epithelial origin of MCC from primitive pluripotent stem cells located in either the epidermis or adnexal epithelium that can differentiate along divergent phenotypes.^[46]^ Other studies have verified that the MCC and SCC components of combined MCC share similar mutational profiles, suggesting a common progenitor possessing bidirectional squamous and neuroendocrine differentiation.^[32]^

#### 5.2.2. Combined MCC derived from 2 separate precursor cells.

Some authors consider their combined MCC cases to be coincidental collision tumors of pure MCC and other skin cancers because there is no clear transition between these 2 tumors histopathologically. A hypothesis of a common carcinogenic influence on the 2 separate precursor cells has been suggested.^[47–49]^ Koba et al reported combined MCC as a collision tumor comprising a rare MCC variant with CK20 negative and CD56/TTF-1 positive immunostaining.^[48]^ Suaiti et al presented a case of combined MCC in the left ear, in which both tumor components metastasized to the same lymph node of the parotid during immunosuppression.^[49]^ This combined MCC is considered a fortuitous collision between the MCC and SCC. A triple-collision tumor with MCC, SCC in situ, and basal cell carcinoma with an unusual CK20 negative and TTF-1 positive immunophenotype has also been reported.^[24]^

## 6. Several subsets arise from different stages of differentiation of common stem cells

### 6.1. Stem cell differentiation

**Figure 1.**
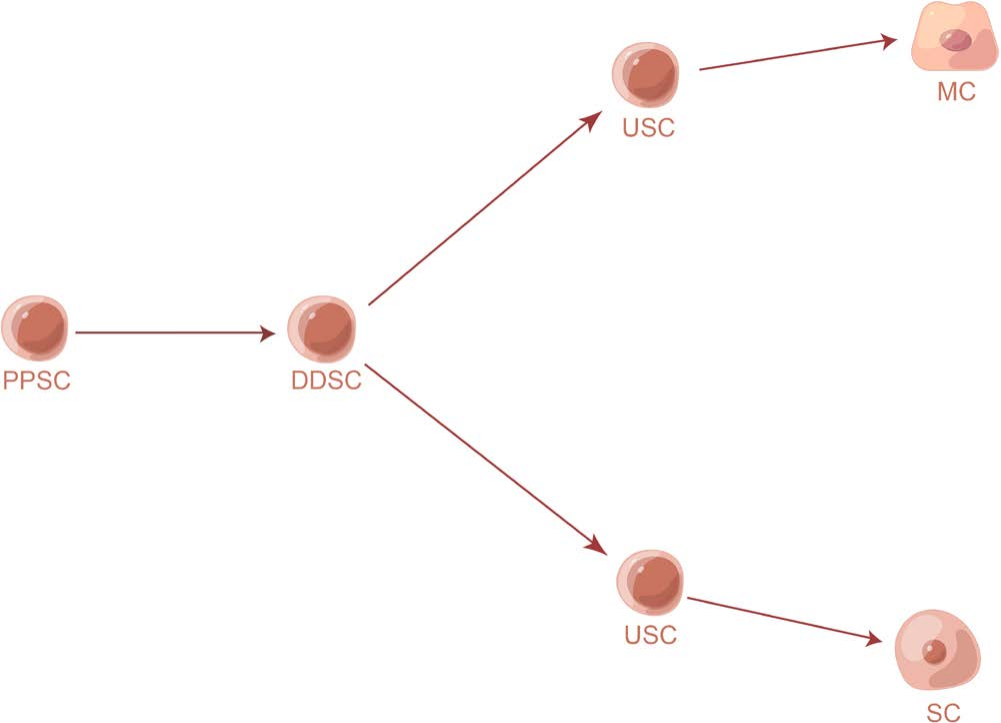
Four stages in the natural history of the stem cell differentiation: PPSC, DDSC, USC, MC, or SC. DDSC = divergent differentiated stem cell, MC = Merkel cell, PPSC = primitive pluripotent stem cell, SC = squamous cell, USC = unidirectional stem cell.

In the recent literature, to clarify the underlying pathomechanism, MCCs are often divided into pure MCPyV-positive, pure MCPyV-negative, and combined MCC. We believe that these subsets may be derived from common stem cells. Based on the existing subsets, it is speculated that there are at least 4 stages in the natural history of stem cell differentiation: primitive pluripotent stem cells, divergent differentiated stem cells, unidirectional stem cells, and Merkel cells (or epidermal/adnexal cells) (Fig. 1).

### 6.2. Pure MCPyV-positive MCC

**Figure 2.**
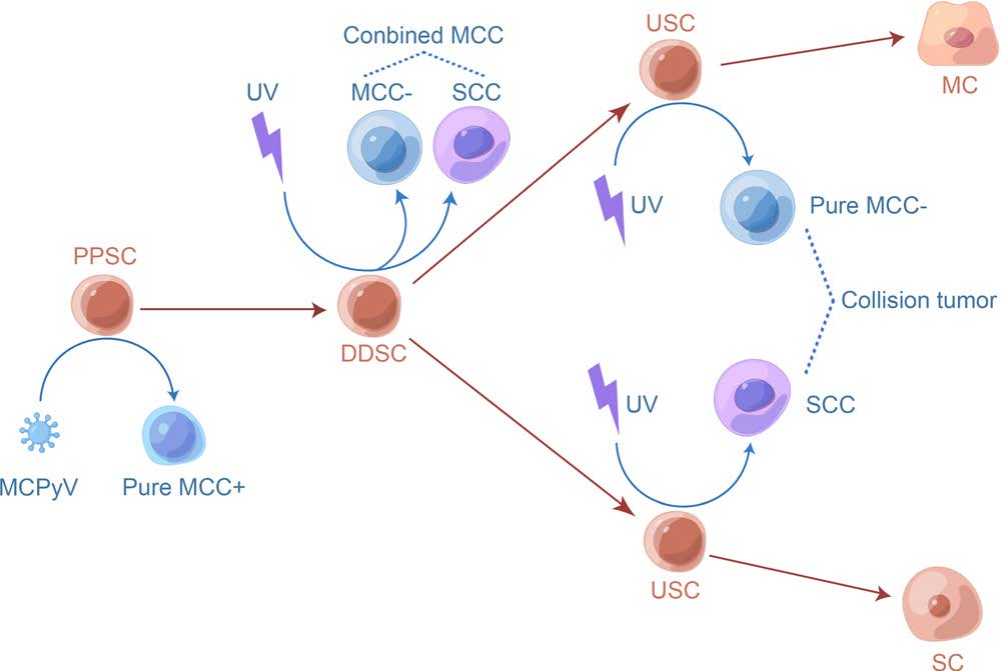
Several subsets arise from different stages of differentiation of common stem cell. In the PPSC stage, MCPyV may integrate into the genome of the stem cell and drive the oncogenesis of pure MCPyV-positive MCC. After the stem cell enters the DDSC stage, accumulated UV-mediated mutations may drive the combined MCC development. Then, stem cells differentiate into the USCs stage, UV-mediated mutations can induce the carcinogenesis of pure MCPyV-negative MCC or SCC. A collision tumor could be formed if 2 kinds of USCs undergo malignant transformation simultaneously at the same anatomical site. DDSC = divergent differentiated stem cell, MC = Merkel cell, MCC− = MCPyV-negative MCC, MCC+ = MCPyV-positive MCC, MCPyV = Merkel cell polyomavirus, PPSC = primitive pluripotent stem cell, SC = squamous cell, SCC = squamous cell carcinoma, USC = unidirectional stem cell, UV = ultraviolet.

The fact that pure MCPyV-positive MCC, associated with viral DNA integration and few mutations, occurs in isolation in the dermis or subcutaneous tissue and lacks epidermal connections has led some authors to postulate that this subset undergoes unidirectional differentiation into transformed progenitor or Merkel cells.^[50]^ Evidence suggests that pure MCPyV-positive MCCs have fewer molecular mutations than MCPyV-negative MCCs. Copy number aberration studies showed fewer genomic changes in pure MCPyV-positive tumors than in the other 2 MCPyV-negative groups.^[31]^ Taking this evidence together prompts the hypothesis that oncogenesis caused by MCPyV integration occurs at the earliest stage of differentiation of primitive pluripotent stem cells prior to UV-induced genetic mutations (Fig. 2).

### 6.3. Combined MCPyV-negative MCC

Many studies have focused on the histogenesis of combined MCC because it contains neuroendocrine and other distinct skin tumors. As mentioned earlier, the theory that the development of both components of the combined MCC from common precursor cells is supported by several experiments. Recently, Kervarrec et al used whole-exome sequencing and found that combined MCC, consisting of SCC and MCC components, showed many mutations shared between the 2 parts, indicating their common ancestry.^[32]^ Moreover, a high tumor mutational burden was evident with an allelic frequency ≥ 10%, and mutation signature analyses confirmed a prominent UV signature in 2 parts of the combined MCC.^[32]^ Accordingly, different components of combined MCPyV-negative MCC can evolve from common stem cells through UV-mediated mutations. The oncogenesis of combined MCC may have appeared later than that of pure MCPyV-positive MCC in the natural history of stem cell differentiation. Stem cells may have the potential for divergent differentiation only at this stage (Fig. 2).

### 6.4. Pure MCPyV-negative MCC

No significant immunohistochemical or molecular differences were observed between the pure and combined MCPyV-negative MCCs. Pasternak et al investigated the expression of 5 immunohistochemical markers (CK20, NF, chromogranin, TTF−1, and CK7) and reported no significant differences between the 2 MCPyV-negative subsets.^[8]^ Carter et al demonstrated that pure and combined MCPyV-negative MCC had similar mutational profiles, harboring deletions and/or mutations in *RB1*.^[31]^ Walsh et al studied global programmed death ligand-1 signals and brisk tumor-infiltrating lymphocytes in 2 MCPyV-negative groups and found no significant differences in either parameter (programmed death ligand-1 or tumor-infiltrating lymphocytes).^[51]^ The shared immunophenotype and genetic mutation profile in pure and combined MCPyV-negative tumors suggest that they are variants of the same entity and serve to separate them from pure MCPyV-positive tumors.^[8]^ Therefore, it was suspected that after the differentiation of stem cells into unidirectional stem cells, which might be driven to develop pure MCPyV-negative MCC through UV-mediated mutations (Fig. 2).

### 6.5. Multiple subgroups of MCCs originate from different stages of differentiation of common stem cells

In the first stage, MCPyV may integrate into the genome of primitive pluripotent stem cells, driving oncogenesis in pure MCPyV-positive MCC. If MCPyV integration does not occur, the stem cells enter the second stage and acquire the ability to undergo multidirectional neuroendocrine and epidermal (or adnexal) differentiation. At this stage, accumulated UV-mediated mutations may drive the development of combined MCC. In the third stage, stem cells differentiate into unidirectional neuroendocrine stem cells, UV-mediated mutations can induce carcinogenesis in pure MCPyV-negative MCC. A collision tumor could be formed if unidirectional neuroendocrine and epidermal (or adnexal) stem cells simultaneously undergo malignant transformation at the same anatomical site through UV-mediated mutations (Fig. 2).

## 7. Conclusion

We reviewed previously published information on the pathogenesis of MCCs and speculated that several subsets arise from different stages of differentiation of common stem cells. However, further studies are required to confirm this hypothesis. Nevertheless, identifying the exact oncogenesis of MCCs not only improves our understanding of this disease but also develops new therapeutic approaches for patients with this lethal cancer.

## Acknowledgments

We would like to thank Editage (www.editage.com) for English language editing and Figdraw (www.figdraw.com) for figures drawing.

## Author contributions

**Conceptualization:** Yaru Dong.

**Data curation:** Yueyang Zhu, Yuan Yin, Fuqiang Li, Zhiyuan Ren, Yaru Dong.

**Formal analysis:** Yaru Dong.

**Funding acquisition:** Yaru Dong.

**Investigation:** Yueyang Zhu, Yuan Yin, Yaru Dong.

**Methodology:** Yaru Dong.

**Project administration:** Fuqiang Li.

**Supervision:** Fuqiang Li.

**Validation:** Yaru Dong.

**Visualization:** Zhiyuan Ren, Yaru Dong.

**Writing – original draft:** Yaru Dong.

**Writing – review & editing:** Yueyang Zhu, Yuan Yin, Fuqiang Li, Zhiyuan Ren.
